# Delphinidin Reduces Glucose Uptake in Mice Jejunal Tissue and Human Intestinal Cells Lines through FFA1/GPR40

**DOI:** 10.3390/ijms18040750

**Published:** 2017-04-05

**Authors:** Jorge Hidalgo, Stefanie Teuber, Francisco J. Morera, Camila Ojeda, Carlos A. Flores, María A. Hidalgo, Lucía Núñez, Carlos Villalobos, Rafael A. Burgos

**Affiliations:** 1 Institute of Pharmacology and Morphophysiology, Universidad Austral de Chile, 5110566 Valdivia, Chile; jihidalgo.godoy@gmail.com (J.H.); steubervolke@gmail.com (S.T.); fjmorera@gmail.com (F.J.M.); camila.ojeda.barria@gmail.com (C.O.); mahidalgo@uach.cl (M.A.H.); 2 Centro de Estudios Científicos (CECs), Avenida Arturo Prat 514, 511046 Valdivia, Chile; cflores@cecs.cl; 3 Instituto de Biología y Genética Molecular (IBGM), Consejo Superior de Investigaciones Científicas (CSIC), 47003 Valladolid, Spain; nunezl@ibgm.uva.es (L.N.); carlosv@ibgm.uva.es (C.V.); 4 Departamento de Bioquímica y Biología Molecular y Fisiología, Universidad de Valladolid, 47003 Valladolid, Spain

**Keywords:** delphinidin, anthocyanins, SGLT1, glucose, GPR40, FFA1

## Abstract

Anthocyanins are pigments with antihyperglycemic properties, and they are potential candidates for developing functional foods for the therapy or prevention of Diabetes mellitus type 2 (DM2). The mechanism of these beneficial effects of anthocyanins are, however, hard to explain, given their very low bioavailability due to poor intestinal absorption. We propose that free fatty acid receptor 1 (FFA1, also named GPR40), is involved in an inhibitory effect of the anthocyanidin delphinidin over intestinal glucose absorption. We show the direct effects of delphinidin on the intestine using jejunum samples from RF/J mice, and the human intestinal cell lines HT-29, Caco-2, and NCM460. By the use of specific pharmacological antagonists, we determined that delphinidin inhibits glucose absorption in both mouse jejunum and a human enterocytic cell line in a FFA1-dependent manner. Delphinidin also affects the function of sodium-glucose cotransporter 1 (SGLT1). Intracellular signaling after FFA1 activation involved cAMP increase and cytosolic Ca^2+^ oscillations originated from intracellular Ca^2+^ stores and were followed by store-operated Ca^2+^ entry. Taken together, our results suggest a new GPR-40 mediated local mechanism of action for delphinidin over intestinal cells that may in part explain its antidiabetic effect. These findings are promising for the search for new prevention and pharmacological treatment strategies for DM2 management.

## 1. Introduction

Intestinal glucose uptake control is a pharmacological target used for the control of hyperglycaemia [1]. Metformin, an effective drug used as a first-line treatment in Diabetes mellitus type 2 (DM2), is accumulated in the mucosa of the intestine increasing glucose turnover and contributing to its antihyperglycaemic effect [2]. In isolated rat jejunal loops, metformin inhibited the glucose-induced short-circuit current, thus reducing the activity of sodium-glucose transporter 1 (SGLT1), and simultaneously increasing the recruitment of glucose transporter 2 (GLUT2) to the apical membrane of the rat jejunum [3]. Since metformin increases intestinal glucose uptake and also lactate production [4,5], this phenomenon can cause intolerance to the treatment [5] and contribute to the development of lactic acidosis [1]. 

Diet is considered a useful non-pharmacological strategy for the control of glycaemia [1]. Recently, considerable attention has been focused on dietary constituents that may be beneficial for the prevention and treatment of diabetes. Diet consumption of polyphenols such as anthocyanins has been associated with a lower risk of DM2 [6,7,8]. 

Anthocyanins, which belong to the flavonoids group, are red or purple plant pigments, present in the form of glycosides in berries. Beyond their anti-oxidant properties, it has been documented that anthocyanins have anti-diabetic properties [9].

Dietary bilberry extract reduces the blood glucose level and enhances insulin sensitivity in type 2 diabetic mice, modulating GLUT4 in white adipose tissue and skeletal muscle, glucose output, and lipid metabolism via AMP-activated protein kinase activation [9]. 

Anthocyanins such as cyanidin-3-glucoside and delphinidin-3-glucoside showed a high-level capability to stimulate insulin secretion from rodent pancreatic β cells in the presence of 4 and 10 mmol/L glucose concentrations [10]. A formulation of anthocyanins or delphinidin 3-sambubioside-5-glucoside decreased fasting blood glucose levels in obese C57BL/6J mice, while in vitro it decreased glucose production in rat liver cells and increased glucose uptake in L6 myotubes [11]. Anthocyanins can be directly absorbed from the gastrointestinal tract, however they show first-pass metabolism (toward anthocyanidins, their aglycone form) by the gut microflora and hence have poor systemic availability [12,13]. In fact, anthocyanins and the sugar-free form, anthocyanidins, are actively transported out of intestinal tissues and endothelia, limiting their bioavailability in plasma [14]. These data suggest that another unknown mechanism might be involved in the control of glycaemia by these pigments. Recently, it has been described that delphinidin can directly induce the release of glucagon-like peptide-1 from enteroendocrine L-cells, and therefore, it could stimulate glucose-dependent insulin secretion in pancreatic β-cells via free fatty acid receptor 1 (FFA1) [15]. In spite of this, patients with DM2 treated with a standardised bilberry extract (36% (*w*/*w*) anthocyanins) had reduced postprandial glycaemia, but showed no significant increase of the GLP-1 level in plasma [16]. Delphidin increases cytosolic free calcium, by releasing Ca^2+^ from intracellular stores and by increasing Ca^2+^ entry in endothelial [17] and T cells [18]. It has been widely demonstrated that the intracellular calcium concentration is involved in glucose transport [19]. However, by using L-type Ca^2+^ channel blockers, an increase in the transport of hexose in rabbit jejunal tissue has been demonstrated, suggesting a more complex role of calcium [20,21]. FFA1 agonist can activate calcium flux via the phospholipase C and L-type Ca^2+^ channel [22], however, the direct effect of these drugs on glucose transport has not yet been assessed. In this paper, we show evidence that delphinidin can reduce glucose uptake in the intestine via stimulation of FFA1, by mechanisms other than incretin release.

## 2. Results

### 2.1. Delphinidin Inhibits Glucose Absorption in Mouse Jejunal

**Figure 1:**
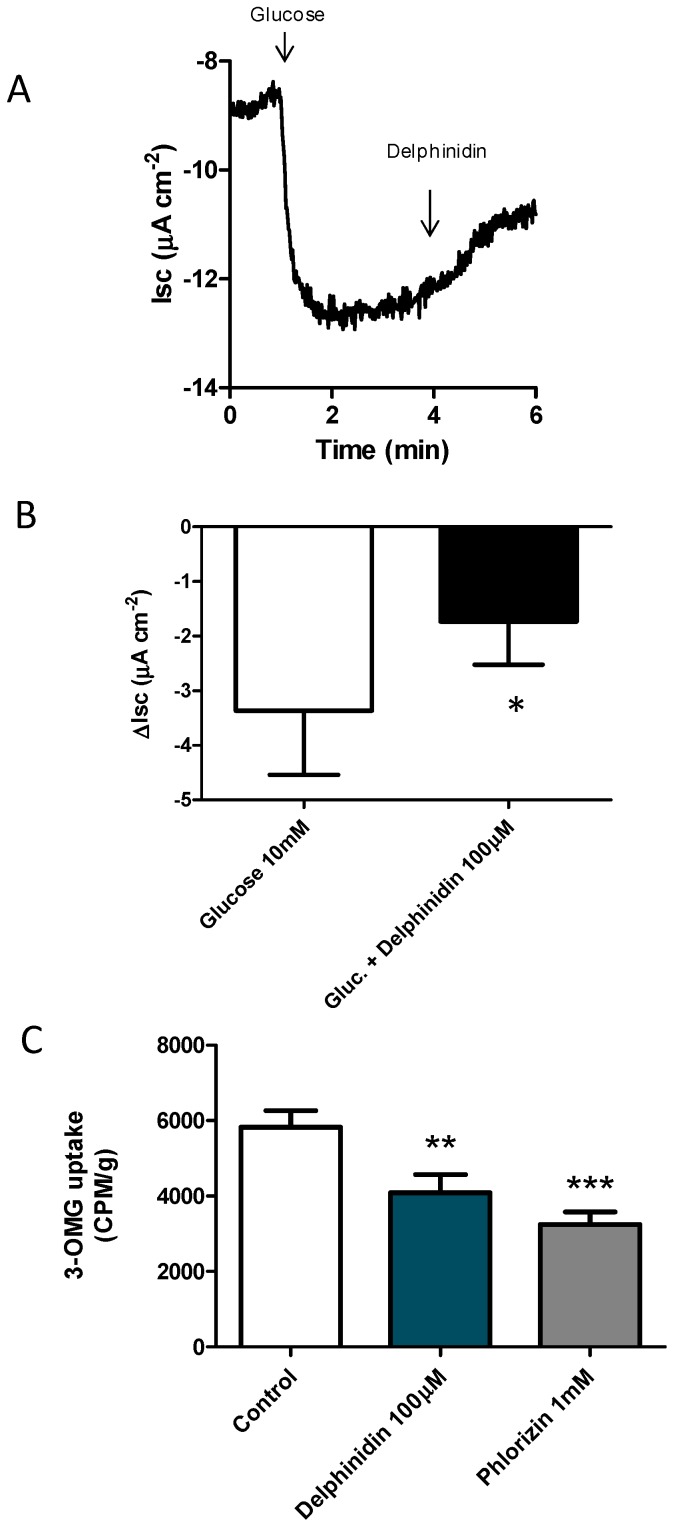
Delphinidin inhibits intestinal glucose absorption. (**A**) Sample tracing showing changes in short-circuit current during an experiment using 10 mM glucose and 100 μm delphinidin; (**B**) Summary of Ussing experiments in Rockefeller mice (RF/J); (**C**) Effect of DMSO 0.2% (control), delphinidin 100 μM, or phlorizin on 3-*O*-methyl-glucose-3H absorption. A glucose uptake is shown as counts per minute per gram of tissue (CPM/g). Bars represent mean ± SEM of at least six different animals. * *p* < 0.05 compared to glucose; ** *p* < 0.01 *** *p* < 0.001 compared to the control.

In order to initially evaluate a possible inhibitory effect of delphinidine on intestinal glucose transport, we decided to study its effect on the electrogenic sodium-coupled glucose transport. Experiments with mouse intestinal preparations in the Ussing chamber (Figure 1A,B) showed that treatment of the jejunal mucosa with 100 μM delphinidin is able to reduce the short-circuit current induced by the addition of 10 mM glucose to the mucosal side. The differences were statistically significant. 

Since the electrophysiological study carried out only allows for the evaluation of the function of the SGLT1 co-transporter, we decided to complement it by performing uptake assays with a radiolabeled glucose analogue in everted sacs of mouse jejunum sections. The results showed that treatment with 100 μM delphinidin significantly inhibited the incorporation of 3-*O*-methyl-glucose [^3^H] (3-OMG) in the mouse intestine (Figure 1C), an effect that is similar to that of phlorizin 1 mM, a pharmacological inhibitor of the electrogenic transport of glucose, which was used as a positive control. 

**Figure 2:**
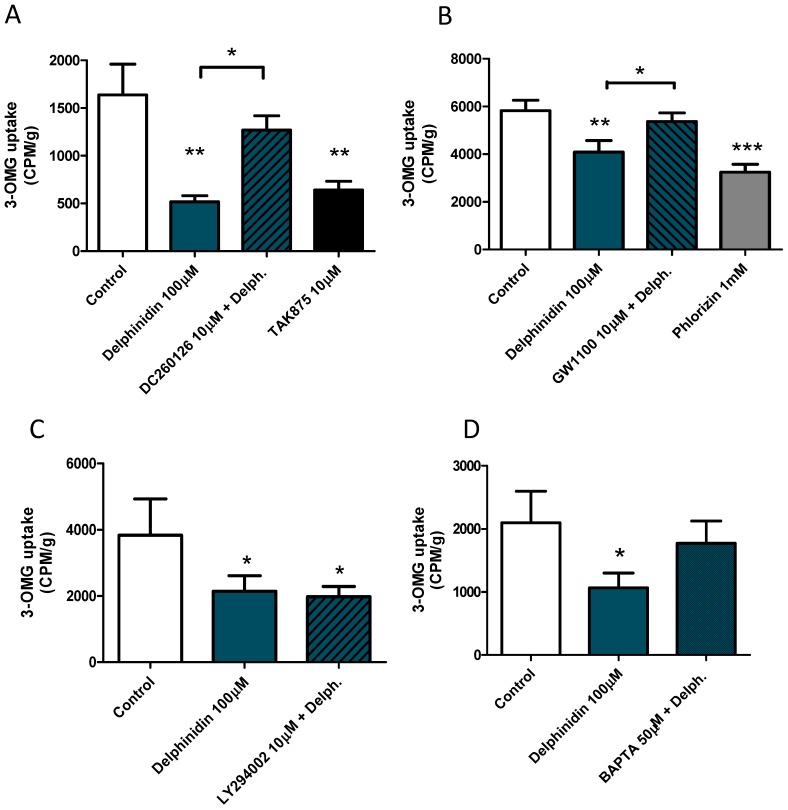
Inhibition of intestinal glucose uptake produced by delphinidin depends on FFA1 and intracellular calcium. Uptake of 3-*O*-methyl-glucose [^3^H] is expressed in counts per minute per gram of tissue (CPM/g). The effects of FFA1 antagonist DC260126 (**A**) and GW1100 (**B**) on delphinidin are depicted. The effect of FFA1 agonist TAK875 on 3-OMG uptake, is shown (**A**). The effect of LY294002, a PI3K Inhibitor, (**C**) or BAPTA-AM (**D**) are shown. Additionally Phlorizin, a SGT1 inhibitor, was used. The bars represent the mean ± standard error of the mean of at least six animals. * *p* < 0.05, ** *p* < 0.01, *** *p* < 0.001, compared with the control. Delph. = 100 μM delphinidin.

In order to determine whether this inhibition of glucose uptake was mediated by the activation of FFA1, we repeated the experiment but added a pre-incubation step of 15 min with pharmacological antagonists of this receptor or vehicle (Figure 2A,B). In these trials, 100 μM delphinidin and the synthetic FFA1 agonist, TAK 875, inhibited the incorporation of glucose significantly (Figure 2A). Tissues pretreated with any of the FFA1 receptor antagonists, DC260126 (Figure 2A), or GW1100 (Figure 2B), interfered with the inhibition of the intestinal glucose uptake produced by delphinidin. In order to study the molecular mechanisms of signaling related to the inhibitory effect of delphinidin on the uptake of glucose in the intestine, we decided to interfere with the activation of the PI3K/Akt kinase pathway through the pharmacological inhibitor of PI3K, LY294002 (Figure 2C), and to interfere with the mobilization of intracellular calcium by the use of the intracellular calcium chelator BAPTA-AM (Figure 2D). In tissues pretreated with 50 μM BAPTA-AM, delphinidin showed no significant inhibition on glucose uptake, whereas in tissues pretreated with the vehicle (0.2% DMSO) or 10 μM LY294002, delphinidin still showed a significant inhibitory effect on glucose uptake.

### 2.2. Presence of FFA1 in Caco-2 and HT-29

**Figure 3:**
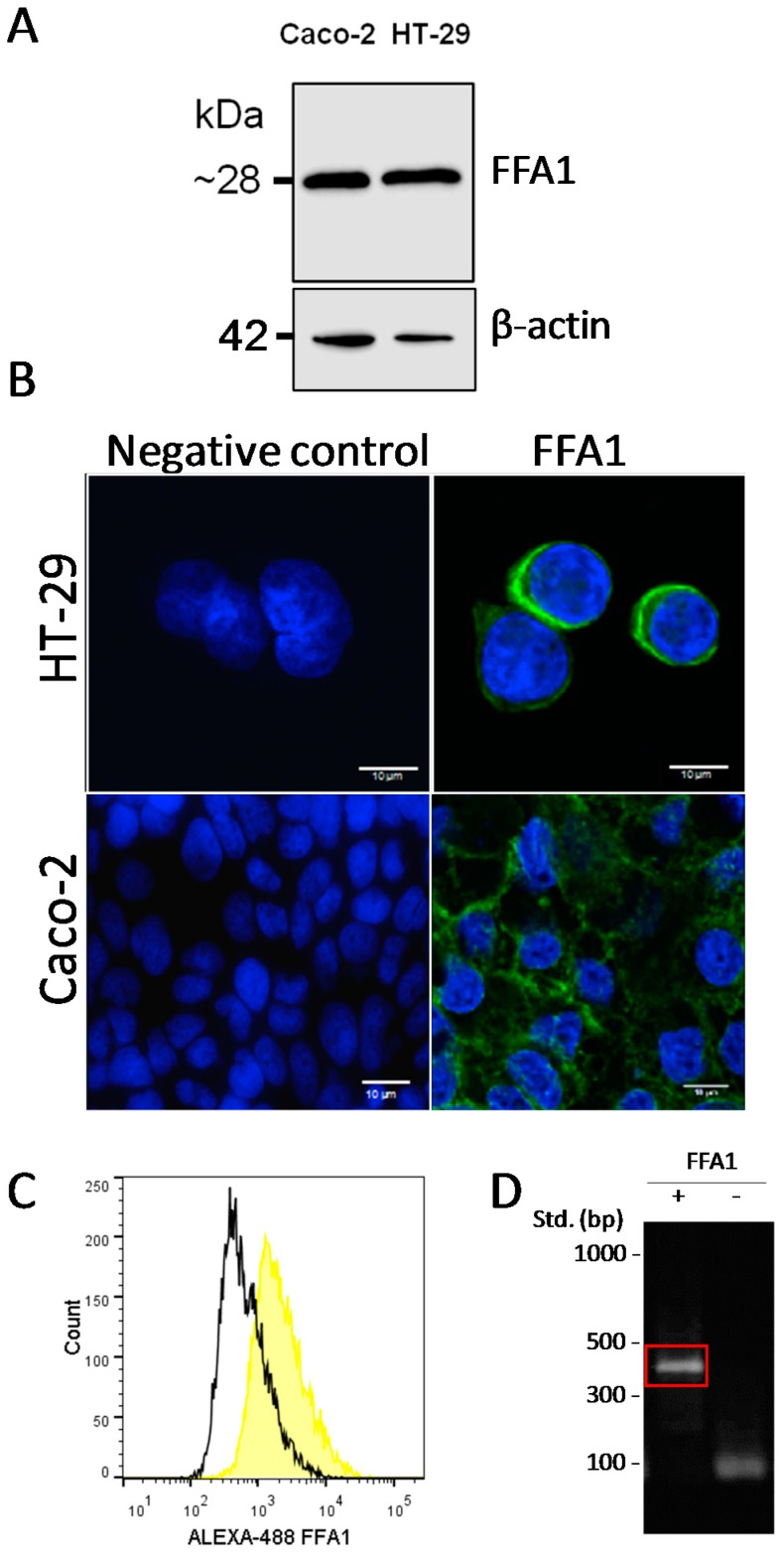
HT-29 and Caco-2 cells express FFA1. (**A**) Immunoblot of FFA1 in HT-29 and Caco-2 cells. A single band near 28 kDa was observed in at least three different assays. The β-actin loading control is shown. Immunofluorescence microscopy of FFA1 in HT-29 Cells and Caco-2 Cells. (**B**) Monoclonal anti-FFA1 antibody was used as the primary antibody, with an Alexa-488-bound fluorescent secondary antibody. Nuclei were stained using Hoechst 33342. Scale bar corresponds to 10 μm; (**C**) Flow cytometry of FFA1 in HT-29 cells. Monoclonal anti-FFA1 rabbit antibody and secondary Alexa-488 bound antibody were used. Results are shown as counts in the Y axis, and fluorescence intensity in the X axis. (Representative experiment from two were carried out); (**D**) qRT-PCR of FFA1 in HT-29 cells. The amplicon for FFA1 is shown in red.

Having observed the inhibitory effect of delphinidin on the intestinal absorption of glucose, and its dependence of calcium and the activation of FFA1, we decided to study in more detail the signaling involved using a cellular model. The presence of the FFA1 receptor was detected in the HT-29 enterocyte-like human cell line by Western blot (Figure 3A) and by immunofluorescence using a confocal microscope (Figure 3B), and was confirmed by immunofluorescence measured by flow cytometry (Figure 3C) and qRT-PCR (Figure 3D).

### 2.3. Delphinidin Induces Intracellular Calcium Release in HT-29 Cells via FFA1

**Figure 4:**
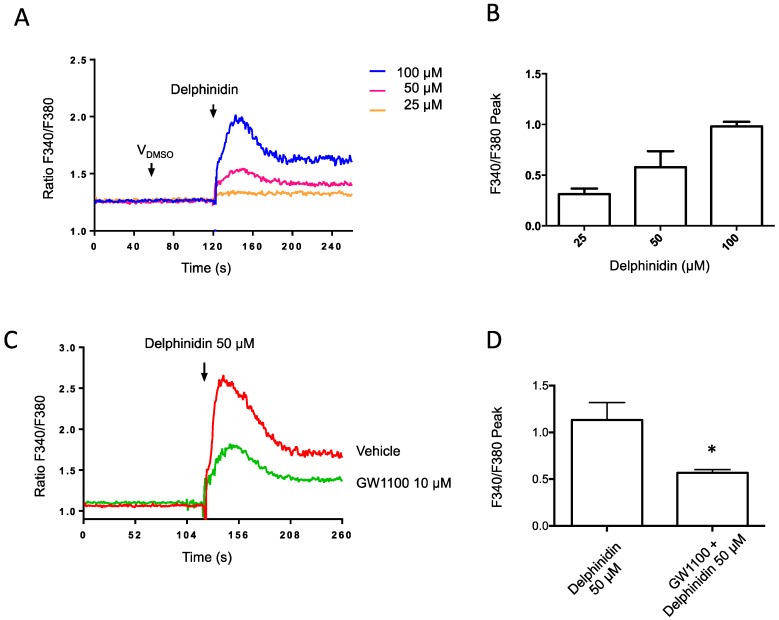
Delphinidin increases intracellular Ca^2+^ concentration in HT-29 Fura-2 AM loaded cells. The changes in cytosolic calcium concentration were evaluated by spectrofluorimetric assays; (**A**) The representative fluorescence record of cytosolic calcium changes induced by delphinidin is depicted; (**B**) Mean data from dose-response experiments in HT-29 cells; (**C**) Intracellular calcium increases induced by delphinidin were inhibited with 10 μM GW1100 (FFA1 antagonist); (**D**) Comparison of the peak observed calcium response with and without FFA1 antagonist treatment. Bars represent mean ± SEM of 3–5 different experiments. * *p* < 0.05.

Using the HT-29 enterocytic line, intracellular calcium measurements in cell populations were carried out with the FURA-2AM fluorescent probe (Figure 4). It was observed that delphinidin was able to induce an increase in intracellular calcium concentrations in a dose-dependent manner (Figure 4B). Upon pre-incubation with the FFA1 antagonist GW1100 (10 μM) for 15 min, the intracellular calcium signal induced by 50 μM delphinidin was diminished (Figure 4C,D)

### 2.4. Delphinidin Induces Intracellular Calcium Oscilations in HT-29 Cells in a Ca^2+^ Store-Dependent Manner 

**Figure 5:**
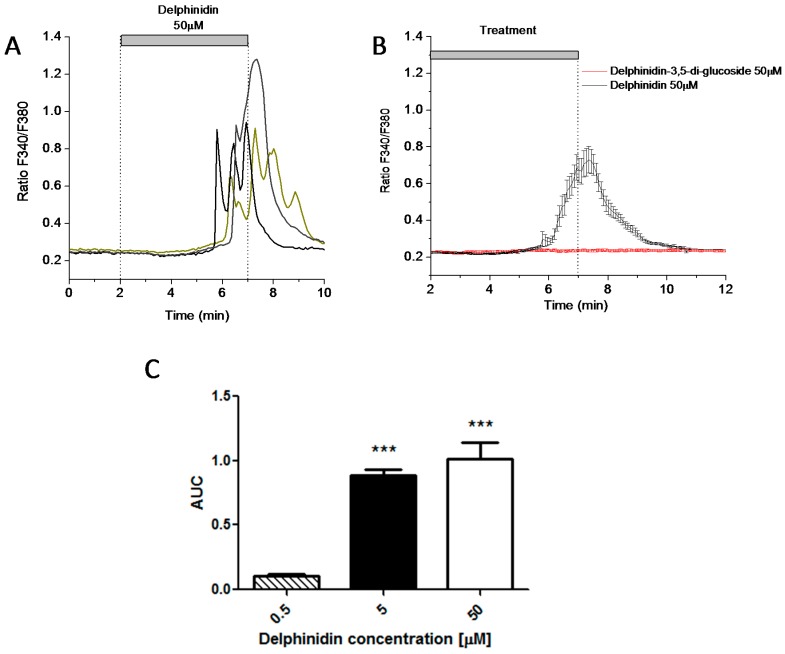
Delphinidin induces intracellular calcium oscillations in HT-29 cells. (**A**) Delphinidin-induced intracellular calcium oscillations at the single-cell level. Each colored line represents calcium recordings of individual cells in the same microscopic field. The horizontal bar depicts the duration of delphinidin perfusion; (**B**) Representative tracing of the mean calcium (± SEM) response of 16 cells treated for 6 min with either Delphinidin-3,5-glucoside 50 μM (**red**) or Delphinidin 50 μM (**black**); (**C**) Area-under-the-curve (AUC) data from dose-response experiments on HT-29 cells. Bars represent mean ± SEM of 16–20 cells. *** *p* < 0.001.

In order to examine the intracellular calcium signals induced by delphinidin in more detail, we decided to perform measurements at the single cell level (Figure 5). At this resolution, it was observed that the intracellular calcium signals produced by delphinidin were actually in the form of oscillations (Figure 5A), that usually started within a few minutes of treatment and which were recorded at concentrations of delphinidin that were even 20 times lower than those effective at inhibiting the transport of glucose analogues in the jejunum from mice (Figure 5C). On the other hand, 50 μM Delphinidin-3,5-glucoside did not induce the effect observed with delphinidin.

**Figure 6:**
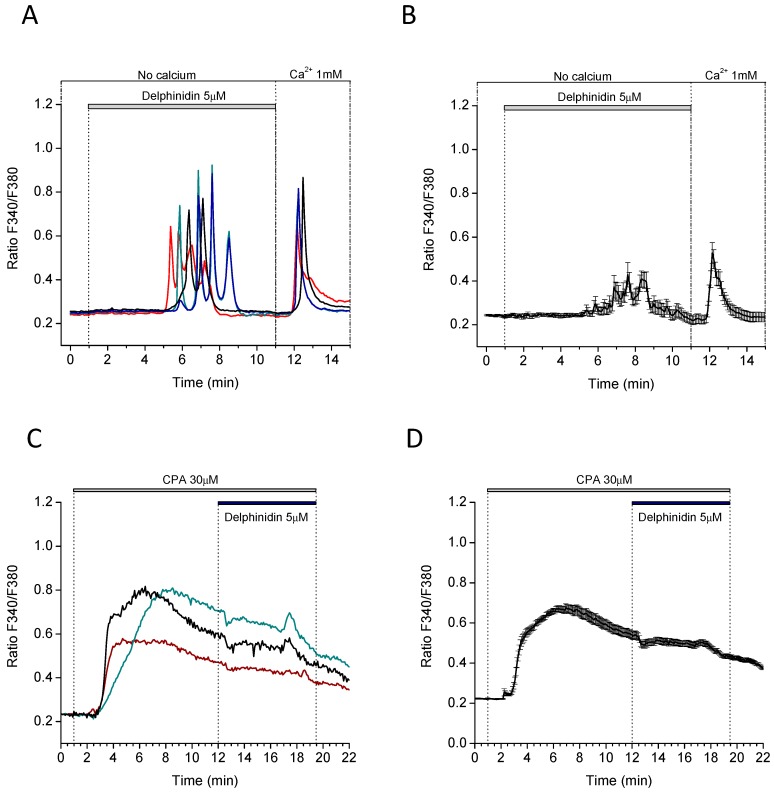
Delphinidin-induces Ca^2+^ store-dependent calcium oscillations and triggers store-operated Ca^2+^ entry in HT-29 Fura-2 AM loaded cells. (**A**) Cells in Ca^2+^-free medium were perfused with 5 μM delphinidin. Each colored line represents the Ca^2+^ concentrations of a single cell. The horizontal bar depicts the duration of perfusion. Before the end of the experiment, extracellular Ca^2+^ containing medium was perfused; (**B**) Mean ± SEM recording from all cells in a representative experiment; (**C**) Cells in HEPES/Ca^2+^ buffer were pre-treated with 30 μM cyclopiazonic acid (SERCA inhibitor) until the stabilization of Ca^2+^ readouts and then the cells were stimulated with delphinidin 5 μM for 7 min. Each colored line represents a recording from individual cells. The horizontal bar depicts the duration of perfusion; (**D**) Mean ± SEM data from all cells in a representative experiment. Data are mean ± SEM of 18 cells.

The characteristic oscillations observed after stimulation with delphinidin could also be reproduced in an extracellular calcium-free medium (Figure 6A), resulting in a smaller average magnitude. It was also observed that the subsequent addition of calcium to the extracellular medium caused a rapid increase in its cytosolic concentration (Figure 6A,B). This calcium influx likely corresponds to the store-operated calcium entry (SOCE) commonly triggered by the emptying of intracellular calcium stores.

After the observation that calcium oscillations can be generated in the absence of extracellular calcium, we decided to confirm if intracellular stores are involved in intracellular calcium oscillations by using 30 μM cyclopiazonic acid, a specific inhibitor of the sarcoplasmic and endoplasmic reticulum Ca^2+^-ATPase (SERCA) of intracellular deposits, that depletes Ca^2+^ stores passively. In this condition, the stimulation with delphinidin was not able to generate the oscillations of intracellular calcium (Figure 6C,D).

### 2.5. Delphinidin Induces Intracellular Calcium Oscilations via FFA1 in Colon Normal Cells

**Figure 7:**
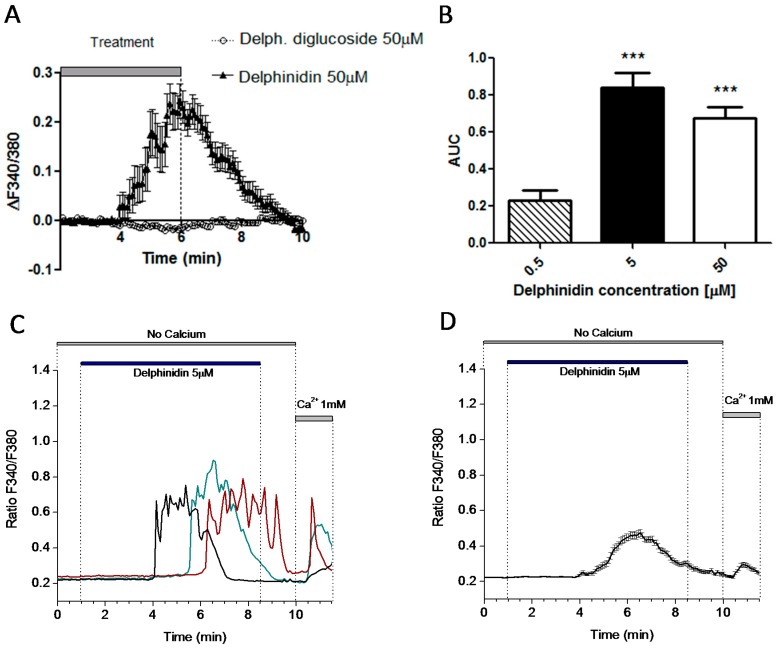
Delphinidin induces intracellular calcium oscillations and store-operated calcium entry in NCM460 Fura-2 AM loaded cells. (**A**) Representative recording (mean ± SEM) corresponding to 19 cells treated with either Delphinidin-3,5-glucoside (black triangles) or delphinidin (empty circles). The vertical symbols represent SEM; (**B**) Area-under-the-curve (AUC) data from dose-response experiments on NCM460 cells; (**C**) NCM460 cells in calcium-free medium were perfused with delphinidin. Each colored line represents calcium concentrations of a single cell. The horizontal bar depicts the duration of perfusion; (**D**) Mean data ± SEM from all cells in a representative experiment from at least 20 cells. *** *p* < 0.001.

Although HT-29 cells are used as a model for human enterocytes, they may behave differently from normal human intestinal mucosa cells [23] as they are derived from adenocarcinoma. With this in mind, we decided to test the same experiment using normal human colon mucosa cells NCM460. Intracellular calcium oscillations, induced by delphinidin but not Delphinidin-3,5-glucoside, also occurred in these cells, this time at minimum concentrations up to 200 times lower than those that had an effect on the uptake of glucose analogues in the mouse jejunum (Figure 7A,B). Delphinidin-induced calcium oscillations could also be seen in calcium-free extracellular conditions, and adding calcium before the end of measurements once again prompted a fast calcium influx (Figure 7C,D).

**Figure 8:**
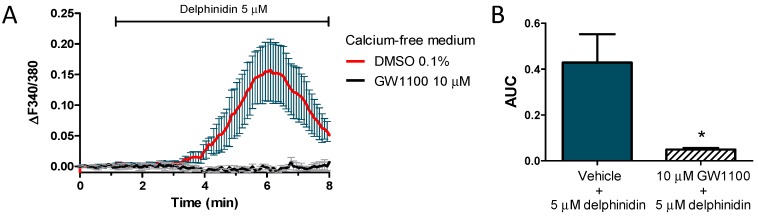
Calcium increase induced by delphinidin in NCM460 Fura-2 AM loaded cells are inhibited by FFA1 antagonist GW1100. (**A**) Temporal course of the ratio F340/380 of HT-29 cells pre-incubated for 15 min with 10 μM GW1100 or vehicle (0.1% DMSO) and then stimulated with 5 μM delphinidin; (**B**) The bar graph indicates the mean area under the curve ± SEM of four experiments. * *p* < 0.05.

To determine if intracellular store-dependent calcium oscillations produced by delphinidin were secondary to the activation of FFA1, calcium measurements were performed on cells exposed to 5 μM delphinidin after being pretreated with the FFA1 antagonist, GW1100 (Figure 8). A significantly lower response to delphinidin was noted in NCM460 cells pretreated with 10 μM GW1100 versus the vehicle (Figure 8B).

### 2.6. Delphinidin Induces Intracellular Calcium Release, cAMP, and Glucose Uptake via FFA1

**Figure 9:**
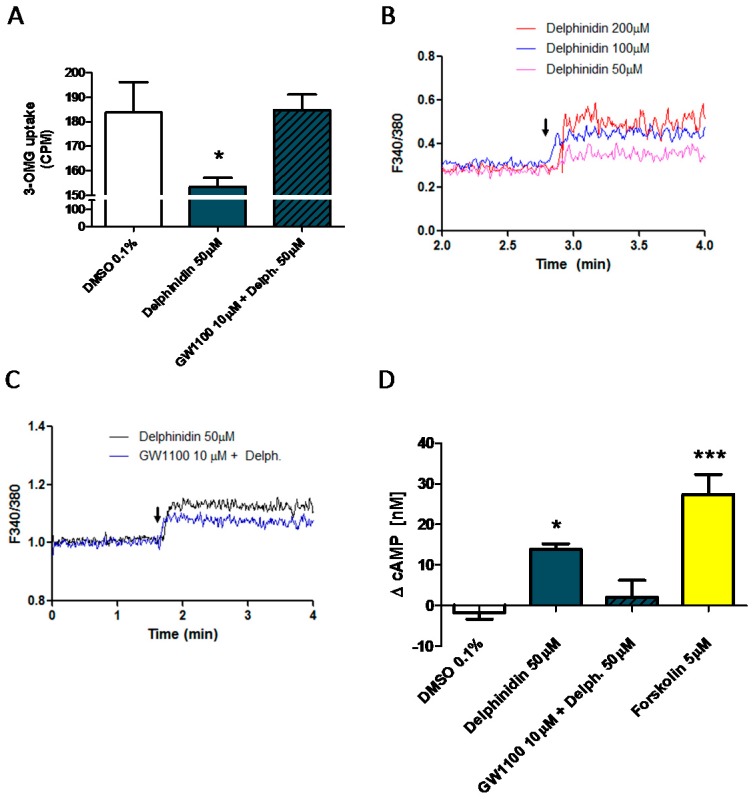
Delphinidin induced inhibition of glucose uptake, intracellular Ca^2+^, and cAMP increase in Caco-2 cell monolayers is reversed by FFA1 antagonist. (**A**) Effect of GW1100 on glucose uptake induced by delphinidin. Each bar represent the mean ± SEM of seven different experiments; (**B**) Effect of delphinidin on Caco-2 Fura-2 AM loaded cells or in the presence of GW1100 (**C**); (**D**) cAMP measurements, bars represent mean ± SEM of four independent experiments. * *p* < 0.05, *** *p* < 0.001.

To assess whether the effects of delphinidin observed on glucose transport in mouse jejunum would also be replicated in a human enterocytic cell line, assays were performed for the uptake of radiolabeled glucose analogs into Caco-2 cells. These cells were selected for glucose uptake assays due to their tendency to spontaneously differentiate into a polarized monolayer of mature enterocytes [23], emulating an epithelial barrier. In cells stimulated with delphinidin at a concentration of 50 μM for 15 min, glucose uptake was significantly reduced compared to the vehicle, except in those cells pretreated with the FFA1 antagonist, GW1100, at 10 μM concentration (Figure 9A).

Finally, in order to further characterize the intracellular signaling induced by delphinidin, we evaluated its effect on the concentration of intracellular cyclic AMP in Caco-2 cells. The luminometric assay demonstrated that incubation with 50 μM delphinidin for 15 min induces a significant increase in cyclic AMP concentration compared to vehicle treatment (Figure 9D). This response was not observed in cells pre-incubated with GW1100.

## 3. Discussion

Polyphenols are known to interact directly with glucose transporters to regulate the rate of glucose absorption [24], reducing electrogenic glucose uptake [25]. Our results suggest that delphinidin reduces electrogenic glucose uptake because its addition decreases the glucose-induced short-circuit current in the jejunum of mice. In the present study, we show new evidence suggesting that delphinidin could directly inhibit the total uptake of glucose in the gut via the FFA1 receptor. This effect of FFA1 on glucose uptake has not been described before, however the roles of FFA1 in glycaemia homeostasis are widely known. The activation of the FFA1 receptor, expressed in L cells and the pancreas, is useful in the control of diabetes via insulin release [26,27]. Also, it has been recently described in the murine GLUTag L cell line that delphinidin can increase GLP-1 secretion via FFA1 [15], which could explain the reduction of glycaemia observed with the administration of anthocyanins in DM2. TAK875 and natural FFA1 agonists such as long-chain free fatty acids, directly stimulate insulin release by the pancreas and incretin release by enteroendocrine cells when arriving at the cell from the vascular side, but not the luminal side [28,29]; these effects require an absorption process prior to stimulating GLP-1 secretion [30]. This is unlikely with delphinidin, because several reports establish that the bioavailability of anthocyanidins is scarce [12,13]. In support of a direct effect of delphinidin on glucose absorption in the gut, we observed that delphidinin reduced the uptake of 3-OMG in mouse everted jejunal ring, and GW1100 and CD260126, two antagonists of FFA1, interfered with the effect of delphinidin. FFA1 activation results in the coupling to the Gαq/11 subunit, enhancing PLC, IP3, and DAG, which stimulates intracellular Ca^2+^ from the endoplasmic reticulum [26,27]. In HT-29 cells, delphinidin induces intracellular Ca^2+^ release characterized by calcium oscillations. The calcium oscillations are initiated when the equilibrium in the basal cytosolic Ca^2+^ level is perturbed. This can occur when the cell senses extracellular stimuli that result in the activation of calcium channels or pumps such as sarco-endoplasmic reticulum Ca^2+^-ATPase (SERCA), that regulate the intracellular Ca^2+^ concentration [31]. Using Ca^2+^ free medium and cyclopiazonic acid, a reversible SERCA blocker, we demonstrated that delphinidin induces intracellular calcium oscillations via store-operated calcium entry SOCE in HT-29. Similarly, other authors have shown that intracellular calcium oscillations are abolished by depleting intracellular calcium stores with cyclopiazonic acid [32]. The SOCE in HT-29 cells has been described before [33], being mediated by the calcium channels ORAI1 and TRPC1 [34]. Agonist-induced Ca^2+^ increases, SOCE, and store-operated currents (ISOC) are largely enhanced in tumor cells, like HT-29 [34], and could limit the interpretation of our results. However, using NCM460 cells (normal human colon mucosa cells), similar intracellular calcium oscillations were observed with delphinidin. Moreover, delphinidin-induced intracellular calcium oscillations have also been described in bovine aortic endothelial cells, and this response was also inhibited by using thapsigargin, a SERCA blocker [17]. 

We observed that delphinidin-3,5-di-glucoside did not induce intracellular calcium oscillations in HT-29 and NCM460, suggesting that anthocyanins, e.g., glycosides of delphinidin, could be a poor FFA1 agonist. In support of this, other authors had previously described that glucosyl anthocyanins did not activate the FFA1 receptors [15]. 

In order to assess the effect of delphinidin on glucose uptake in intestinal cells, we used differentiated Caco-2 cells. Again, delphinidin was able to release intracellular Ca^+2^ and reduce the uptake of 3-OMG via FFA1. Other authors have demonstrated the inhibition of ^3^H-d-glucose uptake in Caco-2 cells using anthocyanin-rich berry extract [35]. Eicosapentaenoic acid (EPA), a natural FFA1 agonist [26,36], also releases Ca^2+^ from the endoplasmic reticulum in colonic epithelial cells, followed by the “store operated” cAMP increase [37]. In a similar way, we demonstrated that delphinidin increases cAMP production in Caco-2 cells via FFA1. There is recent evidence that certain FFA1 ligands can cause the receptor to couple selectively to the Gq or Gs protein [38], with those agonists that can simultaneously activate both pathways being more effective [39]. Previously, this has been described in enteroendocrine cells; however, absorptive intestinal cells and enteroendocrine cells share many common signaling elements, including GPCRs, SGLT1, Ca^2+^, and cAMP [40], and thus could show similar responses upon FFA1 activation.

In spite of these findings, we cannot exclude the possibility that delphinidin may also affect other FFA receptors, since delphinidin could exert dual agonist effects on FFA1/FFA4 in intestinal cells [15]. Moreover, another FFA1 natural agonist, e.g., EPA, can also release Ca^2+^ via FFA4 in human colon epithelial cell lines [41]. 

Altogether, our results suggest that delphinidin can reduce the uptake of glucose in intestinal cells via FFA1, and could explain the reduction of postprandial blood glucose observed with anthocyanins [7,16,42,43,44,45]. Moreover, it would represent a new natural ligand class, beyond the well-known activity of long chain fatty acids. 

## 4. Materials and Methods

### 4.1. Ussing Chamber Experiments

All animal procedures were reviewed and approved by the Institutional Animal Care and Use Committee of the Centro de Estudios Científicos (121DL2-16254, 10-03-2014), according to national regulations. The animal facility of the Centro de Estudios Científicos is accredited by The Association for Assessment and Accreditation of Laboratory Animal Care (Frederick, MD, USA). All animals were fed with 10% kcal% fat (3.85 kcal/g) from Research Diets Inc. (New Brunswick, NJ, USA) which was composed of carbohydrate 67.3%, protein 19.2%, fat 4.3%, and minerals and vitamins 2.1%. Animals of 22 ± 2 g of body weigth, were killed by cervical dislocation and jejunum was isolated and opened longitudinally along the mesenteric border and then rinsed with phosphate buffered saline (PBS). Mouse small intestine samples were selected from jejunal tissue, because duodenal or jejunum show an equivalent pattern of glucose absorption in mice with similar *V*_max_ and *K*_m_ [46]. Two sections of jejunum per animal were mounted in Ussing Chambers and maintained in Ussing buffer (120 mM NaCl, 25 mM NaHCO_3_, KH_2_PO_4_ 3.3 mM, 0.8 mM K_2_HPO_4_, 1.2 mM MgCl_2_, and 1.2 mM CaCl_2_) supplemented with 10 mM d-glucose in the serosal side. The temperature was maintained at 37 °C and the solution was continuously gassed with carbogen containing 5% CO_2_. Once the preparation reached a stable record of electrical parameters, 10 mM d-glucose was added to the mucosal side of the preparation in order to stimulate the Na^+^ coupled d-glucose transport. The effect of 100 μM delphinidin on Na^+^-coupled transport of glucose was determined by its application to the mucosal side of the preparation. The transepithelial electrical potential difference (*V*_te_) was recorded continuously under the current clamp configuration using a VCC MC2 amplifier (Physiological Instruments). The values of the short circuit current (Isc) were calculated from the experimental data using Ohm’s law. Results were expressed as intensity of Isc or the difference (ΔI_sc_) before—after the addition of 10 mM glucose and before—after the addition of 100 μM delphinidin, as described previously [47].

### 4.2. Flow Cytometry Analysis of FFA1 Expression

HT-29 cells cultured in 35 mm dishes were lifted by trypsinization at 37 °C, trypsin activity was stopped by the addition of a complete culture medium, and then cells were pelleted by centrifugation at 600× *g* for 5 min, and afterwards were fixed by 4% paraformaldehyde for 10 min at 37 °C. Then, they were cooled in ice for 1 min, before being centrifuged at 600× *g* for 5 min and resuspended using 90% methanol in PBS (137 mM NaCl; 2.7 mM KCl; 10 mM NaHPO_4_; 2 mM KH_2_PO_4_). Cells were then incubated for 30 min at 4 °C, washed twice with PBS, and treated for 60 min with monoclonal anti-FFA1 antibody (rabbit, Abcam, Cambridge, MA, USA) or Isotype control IgG (rabbit, Abcam) at 4 °C. Afterwards, cells were washed twice with PBS and marked with the anti-rabbit Alexa488-bound secondary antibody for 60 min in darkness. Finally, the cells were washed and then resuspended in PBS. Using a FACSCanto II cytometer (BD Biosciences, San Diego, CA, USA), the cells were displayed as plots of forward light scatter versus side light scatter, and the cell population was identified. The mean fluorescence of Alexa488 was determined from a minimum of 10,000 cells using BD FACSDiva 6.1 software (BD Biosciences).

### 4.3. Glucose Uptake Experiments—Tissue

Methyl-d-Glucose, 3-*O*-[Methyl-^3^H(N)] (lot:657433 Perkin Elmer, Santiago, Chile) uptake was measured in everted jejunal rings [48]. Jejunum from mice was everted and then washed in cold Ringer’s solution (115 mM NaCl, 25 mM NaHCO_3_, 1.2 mM MgCl_2_, 1.2 mM CaCl_2_, 2.4 mM K_2_HPO_4_, 0.4 mM KH_2_PO_4_, pH 7.3) previously bubbled with carbogen (95% O_2_, 5% CO_2_). The sample was then cut into four rings of 0.5 cm length each, which were weighed and then pre-incubated for 15 min in Ringer’s solution constantly bubbled with carbogen at 37 °C with the addition of one of the following: vehicle (0.1% DMSO), a FFA1 antagonist (either 10 μM GW1100 or 10 μM DC260126), or a calcium chelator (50 μM BAPTA). Afterwards, the rings were treated for 15 min with one of the following: 0.2% DMSO (vehicle), 1 mM phlorizin, 100 μM delphinidin, or 10 μM TAK875, before incubation for 2 min in uptake solution (Ringer’s solution bubbled with carbogen plus 10 mM glucose and 0.1 µCi/mL of the isotopic tracer 3-*O*-methyl glucose). Then, the rings were washed with cold Ringer’s solution containing 100 μM cytochalasin B. Tissues were digested in 10% *v*/*v* nitric acid for 24 h and the radioactivity of the supernatant was measured in a Tri-carb 2810 TR liquid scintillation counter.

### 4.4. Glucose Uptake Experiments—Cultured Cells

Methyl-d-Glucose, 3-*O*-[Methyl-^3^H(N)] (lot:657433 Perkin Elmer, Santiago, Chile) uptake was measured in Caco-2 cell monolayers. The Caco-2 cell line is derived from a human colon adenocarcinoma and grows as a monolayer of differentiated polarized cells, showing structural and functional features of small intestinal enterocytes [49,50]. Cultured cells were seeded in 12-well culture plates, at a density of 4 × 10^5^ cells per well. After 24 to 48 h, the culture medium was eliminated and replaced by a differentiation medium (DMEM supplemented with 10% FBS, 1% penicillin/streptomycin, 2 mM sodium butyrate, pH 7.4; modified from Yamashita et al. [51]). After 5 days, the cells were carefully washed and then incubated for 5 min in Ringer’s solution without glucose. Afterwards, the cells were incubated for 15 min in either 0.1% DMSO (vehicle) or 10 μM GW1100, before being treated for 15 min with 50 μM delphinidin or 0.1% DMSO as a negative control. Cells were then incubated for 15 min in cell uptake solution (Ringer’s solution plus 1 mM glucose and 0.3 μCi/mL of the isotopic tracer 3-*O*-methyl glucose). After uptake, cells were exhaustively washed with cold Ringer’s solution plus 10 μM cytochalasin B, then lysed in 1 M NaOH for 24 h. Radioactivity of the supernatant was measured in a Tri-carb 2810 TR liquid scintillation counter.

### 4.5. Western Blot

Cultured Caco-2 and HT-29 cells were lysed in RIPA buffer (50 mM Tris, pH 7.5, 150 mM NaCl, 5 mM EDTA, 1% NP-40, 0.5% sodium deoxycholate, 0.1% SDS, 100 mg/mL PMSF) with the addition of the “complete^®^” commercial protease inhibitor cocktail (Roche) at 4 °C. Protein concentration was determined using the Bradford assay. 50 μg of total proteins were resolved by SDS–PAGE on a 12% polyacrylamide/0.1% SDS gel and were transferred onto a nitrocellulose membrane. After blocking with 5% non-fat dry milk, the membranes were incubated overnight at 4 °C with Anti-FFA1 antibody (rabbit, Abcam) using a 1:1000 dilution. The membrane was washed three times in Tris-buffered saline and was then exposed to 1:5000 anti-rabbit-IgG antibody conjugated with horseradish peroxidase (Cell Signaling, Danvers, MA, USA) for 1 h at room temperature. Blocked antibody solutions were prepared with 1% fat-free milk phosphate-buffered saline (PBS)–Tween 0.1% (PBST), and signals were detected with an enhanced chemiluminescence system. For loading control of FFA1, the antibody was removed by incubation with stripping solution (100 mM 2-mercaptoethanol; 2% SDS; 62.5 mM Tris-HCl, pH 6.7) for 0.5 h at 50 °C with agitation, followed by several washes with TBS-Tween 0.1%. The membrane was then incubated with anti-β-actin antibody (Sigma-Aldrich Quimica Limitada, Santiago, Chile, #A5441) at a dilution of 1:6000, using a procedure similar to that described above. 

### 4.6. Immunofluorescence

Cells were grown on coverslips coated with poly-l-lysine, fixed with 4% paraformaldehyde and permeabilized using 0.3% Triton X-100 for 10 min. Afterwards, they were washed with PBS (137 mM NaCl; 2.7 mM KCl; 10 mM NaHPO_4_; 2 mM KH_2_PO_4_), and were then incubated for 1 h in blocking solution (1% Bovine serum albumin plus 5% non-fat milk in PBS) at room temperature, before being incubated overnight with monoclonal anti-FFA1 antibody (rabbit, Abcam) at 4 °C. On the next day, the cells were washed in PBS and then incubated for 2 h with secondary antibody (Alexa488-bound anti-rabbit antibody). Hoescht 33342 (Invitrogen, Waltham, MA, USA ) was used to stain the cell nuclei. Samples were treated with DAKO Fluorescent mounting medium (Sigma) and then visualized using an inverted Olympus FluoView 1000 confocal microscope (GrupoBios S.A, Santiago, Chile).

### 4.7. Cytosolic Ca^2+^ Monitoring in Cell Suspensions

Fura-2 AM-loaded HT-29 and Caco-2 cells were suspended in HEPES/Ca^2+^ extracellular medium (145 mM NaCl, 5 mM KC l, 1 mM MgCl_2_, 1 mM CaCl_2_, 10 mM HEPES, 10 mM glucose, pH 7.42). Cells were pre-treated for 15 min with either 0.1% DMSO or 10 μM GW100, then stimulated with delphinidin at a concentration of 25, 50, or 100 μM. Fluorescence emission at 509 nm wavelength was recorded after alternate excitation at 340:380 nm using a LS55 thermoregulated spectrofluorimeter (Perkin-Elmer, Waltham, MA, USA ).

### 4.8. qRT-PCR of FFA1 in HT-29 Cells

Total RNA extraction was done with the commercial kit E.Z.N.A. Total RNA Kit I (Omega Bio-Tek, Atlanta, GA, USA). Then the cDNA was made from 1 μg of total RNA with reverse transcriptase M-MLV (Promega, Madison, WI, USA). Finally, a conventional PCR of 40 cycles (denaturation 30 s at 95 °C, alignment 30 s at 55 °C, and elongation 1 min at 72 °C) was performed with a GoTaq Green Master Mix (Promega). The primers were designed against FFA1 sense: CTGGTCTACGCCCTGAACCT; antisense: GAGCCTCCAACCCAAAGACC.

### 4.9. Cytosolic Ca^2+^ Imaging in Adhered Cells

Intracellular calcium was measured by fluorescence microscopy as described by Núñez et al. [52]. Cultured cells were seeded on 12 mm cover glasses. After 24 h, cells were loaded with 4 μM Fura-2 AM for 60 min in a HEPES/Ca^2+^ extracellular medium (145 mM NaCl, 5 mM KCl, 1 mM MgCl_2_, 1 mM CaCl_2_, 10 mM HEPES, 10 mM glucose, pH 7.42). For experiments with the FFA1 antagonist, the final 30 min of loading also included pre-incubation with 10 μM GW1100. Afterwards, the cells were placed on a platform attached to the stage of an inverted Zeiss Axiovert microscope equipped with an OrcaER Hamamatsu digital camera, where they were constantly perfused with HEPES/Ca^2+^ at 37 °C. For calcium-free extracellular medium experiments, the perfusion was then switched to HEPES calcium-free extracellular medium (145 mM NaCl, 5 mM KCl, 1 mM MgCl_2_, 10 mM HEPES, 10 M glucose, 0.5 mM EGTA). Cells were epi-illuminated alternately at 340 and 380 nm wavelengths, and the light emitted above 520 nm was recorded every 5 s.

### 4.10. cAMP Measurements

Caco-2 cells were seeded at a density of 4000 cells per well in flat clear-bottom poly-d-Lysine treated 384-well microplates (Corning, New York, NY, USA). On the next day, cells were treated with 10 μM GW1100 (FFA1 antagonist) or 0.1% DMSO (vehicle) for 15 min, and were then treated with delphinidin 50 μM, DMSO 0.1%, or Forskolin 5 μM (as positive control) for 15 min. Cytosolic cAMP concentrations were determined using the commercial “cAMP-Glo^®^A” luciferase-based assay kit (Promega), following the manufacturer’s instructions, including treatment with phosphodiesterase inhibitors (100 μM RO 20-1724 and 100 μM IBMX in DMEM culture medium without FCS). Finally, the luminiscence was measured in a Luminoskan Ascent microplate luminometer (Thermo Scientific, Waltham, MA, USA).

### 4.11. Statistical Analysis

Normality was checked by the *Kolmogorov-Smirnov* test. For comparisons between two groups, the Student’s *t* test was used. For more than two groups, analysis of variance was performed, followed by the Newman-Keuls multiple comparisons test. For correlated experimental data, randomized block ANOVA was used, as suggested by Lew [53].

## Abbreviations

SGLT1	Sodium-glucose cotransporter1
DM2	Diabetes mellitus type 2
FFA1	Free fatty acid receptor 1
3-OMG	3-*O*-methyl-glucose [^3^H]
